# How fast and how well the Omicron epidemic was curtailed. A Guangzhou experience to share

**DOI:** 10.3389/fpubh.2022.979063

**Published:** 2022-12-21

**Authors:** Wenfeng Cai, Zifeng Yang, Jingyi Liang, Zhengshi Lin, Yu Ma, Chun Chen, Yan Li, Yongming Li, Zhitong Mai, Kailin Mai, Xuetao Kong, Xingyi Liang, Qianying Li, Chuanmeizi Tu, Canxiong Chen, Chitin Hon, Pengzhe Qin, Ke Li, Xiaoning Li, Yutian Miao, Xuexing Liu, Wenda Guan, Zhiqi Zeng, Wanli Qiu, Wei He, Lin Zhang, Zhicong Yang, Nanshan Zhong

**Affiliations:** ^1^Institute of Public Health, Guangzhou Medical University & Guangzhou Center for Disease Control and Prevention, Guangzhou, Guangdong, China; ^2^National Center for Respiratory Medicine, State Key Laboratory of Respiratory Disease & National Clinical Research Center for Respiratory Disease, Guangzhou Institute of Respiratory Health, The First Affiliated Hospital of Guangzhou Medical University, Guangzhou, Guangdong, China; ^3^Guangzhou Laboratory, Bio-Island, Guangzhou, China; ^4^State Key Laboratory of Quality Research in Chinese Medicine, Macau Institute for Applied Research in Medicine and Health, Macau University of Science and Technology, Macao, Macau SAR, China; ^5^Guangzhou Key Laboratory for Clinical Rapid Diagnosis and Early Warning of Infectious Diseases, Guangzhou, China; ^6^Macau Institute of Systems Engineering, Macau University of Science and Technology, Macao, Macau SAR, China; ^7^Guangdong Center for Disease Control and Prevention, Guangzhou, Guangdong, China; ^8^Shunde Urban and Rural Planning Information Research Center, Foshan, Guangdong, China

**Keywords:** COVID-19, public health, control measure, epidemiological investigation, health code

## Abstract

**Introduction:**

SARS-CoV-2 has ravaged the world and undergone multiple mutations during the course of the COVID-19 pandemic. On 7 April 2022, an epidemic caused by SARS-CoV-2 Omicron (BA.2) variant broke out in Guangzhou, China, one of the largest transportation and logistical hubs of the country.

**Methods:**

To fast curtained the Omicron epidemic, based on the routine surveillance on the risk population of SARS-CoV-2 infection, we identify key places of the epidemic and implement enhanced control measures against Omicron.

**Results:**

Transmission characteristics of the Omicron variant were analyzed for 273 confirmed cases, and key places involved in this epidemic were fully presented. The median incubation time and the generation time were 3 days, and the reproduction number Rt was sharply increased with a peak of 4.20 within 2 days. We tried an all-out effort to tackle the epidemic in key places, and the proportion of confirmed cases increased from 61.17% at Stage 2 to 88.89% at Stage 4. Through delimited risk area management, 99 cases were found, and the cases were isolated in advance for 2.61 ± 2.76 days in a lockdown zone, 0.44 ± 1.08 days in a controlled zone, and 0.27 ± 0.62 days in a precautionary zone. People assigned with yellow code accounted for 30.32% (84/277) of confirmed COVID-19 cases, and 83.33% of them were detected positive over 3 days since code assignment. For the districts outside the epicenter, the implementation duration of NPIs was much shorter compared with the Delta epidemic last year.

**Conclusion:**

By blocking out transmission risks and adjusting measures to local epidemic conditions through the all-out effort to tackle the epidemic in key places, by delimiting risk area management, and by conducting health code management of the at-risk population, the Omicron epidemic could be contained quickly.

## Introduction

SARS-CoV-2 has ravaged the world and undergone multiple mutations during the course of the COVID-19 pandemic (1). The latest Omicron variant B.1.1.529.1, a variant of concern (VOC), was first identified in South Africa in November 2021; it rapidly replaced the Delta variant and predominantly circulates worldwide at present with an R0 of >7.0 (2, 3). The World Health Organization's (WHO) COVID-19 Weekly Epidemiological Update on 5 April 2022 (4) reports over 9 million new cases and over 26,000 new deaths during the week of 28 March to 3 April 2022 across the six WHO regions. Among the 417,147 sequences uploaded to GISAID with specimens collected in March 2022, 416,175 (99.8%) were Omicron, the relative proportion of Omicron lineage BA.2 has increased from 85.38% at Week 11 to 93.6% at Week 13 in 2022.

Compared to other VOCs (especially the Delta variant), a marked degree of mutations, enhanced transmissibility, and immune evasion have been apparently observed (5). It can cause reinfection in a person who has recovered from a previous infection, as well as breakthrough infections in vaccinated populations. In fact, the highly transmissible Omicron has caused widespread infections within a short period of time somewhere in the world, resulting in a sudden increase in large numbers of cases and case fatalities (6). These characteristics bring new difficulties and challenges to the prevention and control of the outbreak. It is evident that if the epidemic was not contained at the initial stage, it is likely to cause the extension of the epidemic, ultimately paying a much higher price to control, such as requiring excessive manpower and material resources, overwhelming medical and health systems, and even losing more lives tragically.

On the other hand, the COVID-19 pandemic has been lingering for more than 2 years, and a return to normal life has become a strong desire. Thus, implementation of epidemic prevention and control measures must be scientifically and timely adjusted according to current epidemiology and specific conditions of different countries, as such, managing to strike a balance between strict prevention and control measures and minimizing the impact on people's normal life is an important issue of the moment (7, 8). Since the clinical symptoms of Omicron are milder than those of Delta (predominantly with upper respiratory tract infection symptoms) (9, 10), some countries/areas have loosened their control measures based on the high infection rate and vaccination rate (11). China is the world's second most populous developing country; up to 19 April 2022, the nationwide vaccination rate (three doses) is approximately 51% (12), therefore, precise and strict preventive and control measures are necessary to buy more time for people at high risk to be vaccinated.

An epidemic caused by the Omicron variant broke out on 8 April 2022 in Guangzhou, China. Guangzhou has a resident population of over 18 million; it is the transportation hub of southern China and is one of the largest logistical hubs in the country. Based on our experience in controlling Delta's outbreak in the midyear of 2021 (13) and the epidemiological characteristics of Omicron, we quickly adopted precise control measures to contain the outbreak within 18 days and minimized the impact on society. Here, we present the Omicron epidemiological characteristics and share our experience in implementing control measures to contain the epidemic.

## Materials and methods

### Source of data

Data of demographics and epidemiological investigation of confirmed cases from 8–26 April 2022 were extracted from the collection of the Guangzhou Center for Disease Control and Prevention on 1 May, including patients' age, sex, vaccination status, residential district, history of close contact with confirmed cases, the number of risk population being tested, total infected cases diagnosed by real-time reverse transcriptase-polymerase chain reaction (RT-PCR) assay, health code assignment, confirmed cases in isolation, and the laboratory confirmation date.

### Routine surveillance on the risk population of SARS-CoV-2 infection

Since the outbreak of the Delta variant, we set up the routine surveillance on the risk population for nucleic acid testing, which was divided into three groups: (A) compulsive nucleic acid testing group; (B) high-risk industries and population group; and (C) potential risk groups and risk areas. The definitions and detailed nucleic acid testing requirements of these groups are described in Supplementary Table S1. Taking potential risk groups and risk areas as an example, a screening nucleic acid test (NAT) was taken once a week on 25% of the population in the group, and the entire population was tested once a month.

### Identification and management of key places

**Figure 1:**
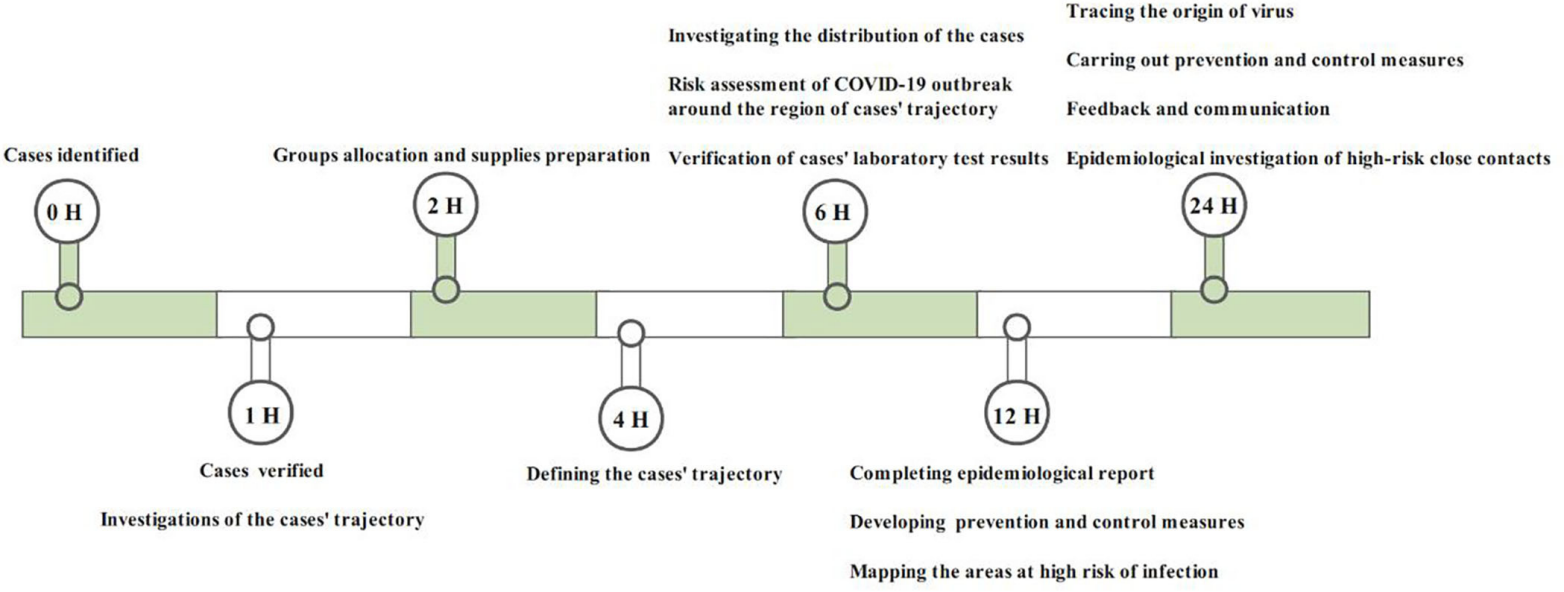
“24-h” epidemiological survey protocol.

**Figure 2:**
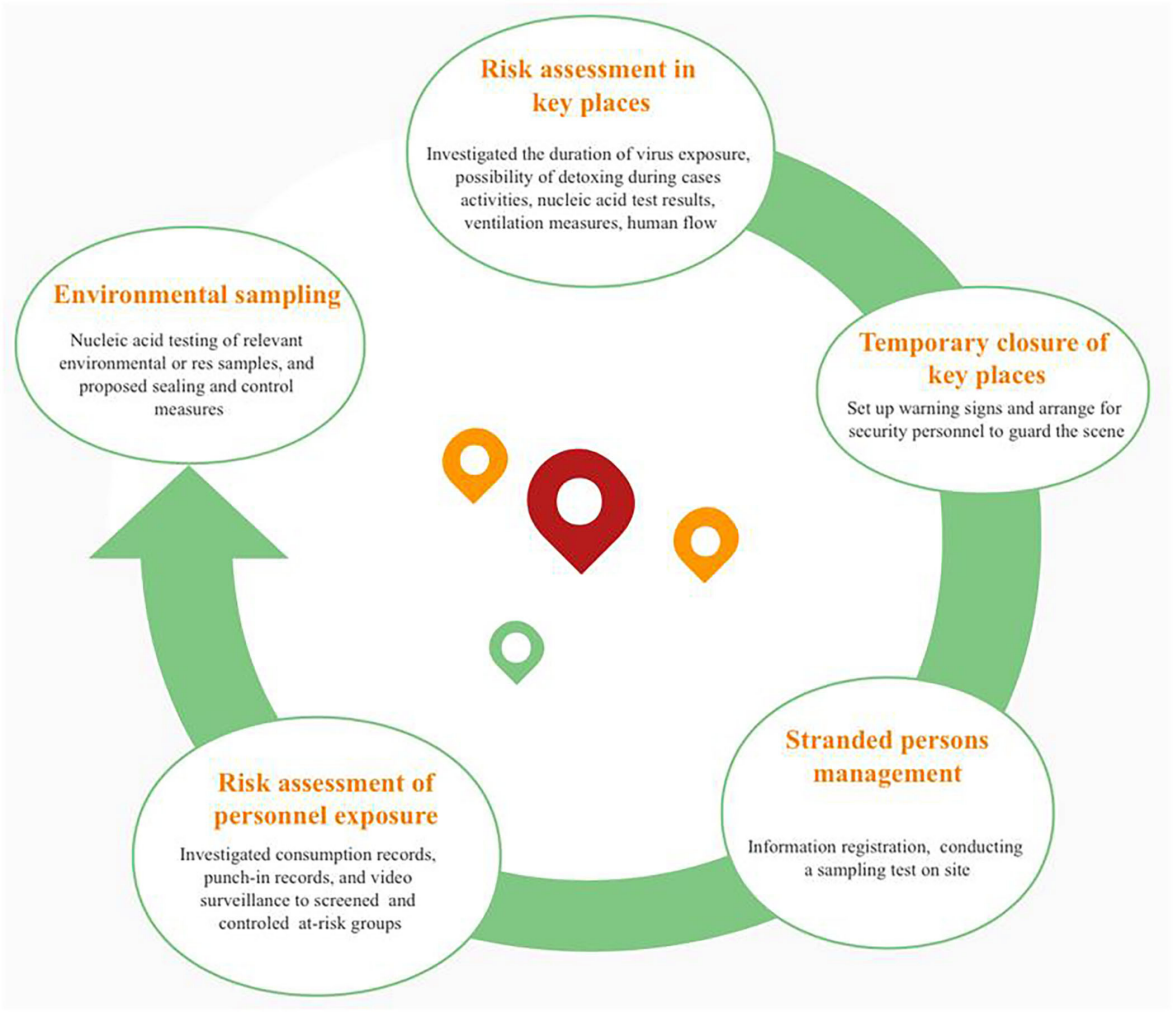
Identification and management of key places.

With the joint effort of Guangzhou government departments, a “24-h” epidemiological survey protocol (Figure 1) was compiled to identify the risk populations and key places rapidly. According to this protocol, key places were defined based on where the confirmed cases stayed 2 days prior to the onset of illness. Subsequently, based on the information on the length of stay and frequency of confirmed cases visited, the completeness of close contact personal information, and the environmental ventilation of the premises, we further divided certain areas into high-, middle- and low-risk key places (Supplementary Table S2). Management of high-risk key places was one of the critical tactics in the overall anti-epidemic work, which included risk assessment, temporary closure, nucleic acid testing and registration information of stranded persons, risk assessment of personnel exposure, and environmental sampling and disinfection (Figure 2).

### Enhanced control measures against Omicron

According to the judgment on the epidemic trend, we literally divided the whole epidemic into four stages (8–9 April, 10–12 April, 13–15 April, and 16–18 April 2022). Due to the higher transmissibility of Omicron, we thus modified and improved our previous prevention and control measures for the Delta variant (Table 1). The following three points were made:

1) In terms of the management of regional control, we divided Baiyun District (the epicenter of the outbreak) into the following three areas: lockdown zone, controlled zone, and precautionary zone. In the lockdown zone, people were quarantined at home and living with the help of volunteers; in the controlled zone, a number of grids had been set up, with no movement between grids permitted; and in the precautionary zone, people who entered and left the area must have the certificate of negative nucleic acid testing within 48 h. Classification of the three areas was based on the confirmed cases' trajectory and is summarized in Table 1.
2) People with yellow health code were required to have three NATs taken within 7 days (each test taken at least 24 h apart). When a negative result is obtained, the person's health code would turn green, but if the test was not taken as required, the yellow code would be assigned again. To avoid cross-infection, people with green and yellow codes had to be tested separately.
3) In addition to the regular NATs, we also introduced antigen testing in the precautionary zone.

**Table 1:** The enhanced intervention in the 2022 Guangzhou Omicron epidemic.

		**2022 Omicron**	**2021 Delta**
Regional control measures	Region designation	• Set up lockdown zones, controlled zones, and precautionary zones. • Divide lockdown and controlled zones as grids.	• Set up lockdown zones and control zones
	Basis of region designation	• Lockdown zones refer to multiple grids where infected people live or work and where they had stayed 4 days before the symptom onset. • Controlled zones refer to grids where a certain risk of transmission has been assessed^a^. • Precautionary zones refer to the blocks and towns where the lockdown zones and controlled zones are located.	• Lockdown zones refer to neighborhoods where infected people live or work. • Control zones refer to neighborhoods where a certain risk of transmission has been assessed.
	Basis of lifting control measures	• Lift lockdown or controlled zones (1) There are no new cases or asymptomatic infections in the area in the past 14 days; (2) The last close contact in the area has been isolated for more than 10 days since the last exposure or more than 4 days since the centralized transportation, and the nucleic acid test is negative; (3) Two days before unsealing or decontrol, all personnel in the area completed a round of nucleic acid screening and the results were negative. • Lift precautionary zones When all sealed and controlled areas in the prevention area have been removed.	• Lift community (village) closed management. There was no new infection within 14 days, the cases and close contacts were effectively controlled.
	Regional management	Lockdown zones: • All residents are not allowed to leave their dwelling units during the lockdown. • All residents shall take a nucleic acid test within 24 h, then get tested every day in the first 7 days, and then every 2–3 days in the next 7 days during the 14-day virus incubation period. • Single-sample tube nucleic acid testing is carried out for all. • “1 + 5 + 3 + 9 + N” working mechanism^b^	Lockdown zones: • All residents are not allowed to leave their residential buildings for quarantine. • PCC (Primary Close Contacts) conduct daily nucleic acid testing from days 1 to 7, day 10, and day 14 while quarantining. • SCC (Secondary Close Contacts) conduct nucleic acid testing on day 1, 4, and 7 while quarantining. • Other people conduct mass testing.
		Controlled zones: • Residents from one grid cannot travel to the other. • Residents shall conduct nucleic acid testing once within 24 h, every day for the first 3 days, and then every 2–3 days in the following period. • “1 + 5 + 3 + 9 + N” working mechanism	Control zones: • Allowed to enter but not allowed to leave. • PCC conducts daily nucleic acid testing from days 1 to 7, day 10, and day 14 while quarantining. • SCC conducts nucleic acid testing on day 1, day 4, and day 7 while quarantining. • Other people conduct mass testing.
		Precautionary zones: • People who enter and leave the area must have a certificate of negative nucleic acid testing within 48 h. • Carry out a full staff nucleic acid testing within 24 h, and the follow-up arrangement is according to the specific arrangement of each district.	People who are self-quarantined at home should conduct nucleic acid testing according to the general screening.
Yellow health code	Basis of yellow health code assignment	• People who have visited key places^c^ during a particular period^d^ and stayed more than 1 h. • People who have not undergone nucleic acid testing as required.	• People who stayed for over 1 h within 500 m of key places at the same time or in the key place at the same time as the infected up to 17 Jun 2021.
	Management of yellow health code	• Conduct a 7-day home health monitoring and avoid unnecessary travel. • Get three PCR tests taken within 7 days (on the first, third, and seventh days, respectively), with each test taken at least 24 h apart. • Health code turns yellow again when PCR tests not taken as required. • Set designated testing site for yellow code holders only.	• Conduct home health monitoring. • Get three PCR tests taken within 7 days (on the 1st, 3rd, and 7th days, respectively).
Testing strategy		• Nucleic acid testing • Antigen testing^e^	Nucleic acid testing

^a^The assessment criteria include the sojourn time and health situation of the confirmed cases and the ventilation of the certain place, and so on.

^b^“1 + 5 + 3 + 9 + N” working mechanism: Each task force consists of one high-ranking official as the grid leader, five mid-ranking officials as the main staff, as well as three public security policemen, three medical staff, nine security personnel, as well as party members, property management personnel and volunteers, to strengthen the management of three classified zones.

^c^Key places refer to the places where multiple confirmed cases, including asymptomatic infections, have visited or the confined spaces or poorly ventilated areas, where any infections have been found, including the surroundings within 250 m in diameter.

^d^A particular period refers to from the moment a confirmed case once stayed to an hour later, including 2 days before symptom onset until to be quarantined.

^e^Antigen testing: first used in Baiyun District, and then used in other districts according to the guidance of the National Health Commission that three categories of people are able to take an antigen test: (1) people who visit grassroots medical facilities after feeling suspicious respiratory symptoms or having a fever within 5 days; (2) people undergoing centralized or home isolation; and (3) residents in need of such tests due to personal reasons.

### Statistical analyses

Normal distribution data, such as the interval between control and testing positive in the delimited risk area, were presented as mean (±standard deviation). Qualitative information, such as the source of case findings, was presented as percentage or frequency. The Spearman rank test was performed in the comparison of interval calculation between two epidemics. All of the tests were two-tailed, and a value of *P* < 0.05 represented statistical significance. The statistical analysis was conducted using statistical R version 4.0.2. We applied a Bayesian framework to estimate the Rt value of the Omicron strain, which used a Gamma distributed prior, conjugated to the Poisson likelihood, and obtained an analytical formulation of the posterior distribution of Rt. To maintain the accuracy of the prediction and without hiding the underlying time trend, Rt values were estimated over a 7-day moving window.

## Results

### Demographic characteristics of the Omicron variant infection in Guangzhou

A total of 277 cases were diagnosed in this epidemic. Among the mentioned cases, 251 (90.6%) were identified as confirmed cases, whereas 26 (9.39%) were as asymptomatic cases. Demographic characteristics of the confirmed cases are summarized in Table 2. The median incubation time and generation time were also 3 days, which means that the transmission is quick and insidious. The median age was 33 years (IQR:23.5, 43), and the male patients accounted for 45.78%. Infectious cases aged 15–44 years accounted for the majority of (69.96%) of the total cases. A total of 239 (87.55%) cases had received 2 or 3 doses of inactivated vaccine, and a total of 25 cases (9.15%) had not been vaccinated. Compared to the cases discovered from mass testing (1.10%), risk population (3.66%), and fever clinic (1.83%), most infectious cases were found among close contacts (56.41%) and in the delimited risk areas (36.26%) (Table 2).

**Table 2:** Demographic characteristics of the subjects infected with the SARS-CoV-2 Omicron variant in Guangzhou, 2022.

**Characteristics**	**No. (%)**
Total	273^a^
Male—no. (no./total no.%)	125 (45.78)
Age (years)-median (IQR)	33 (23.5,43)
Incubation time-median (Range)	3.00 (1.00, 6.00)
Generation time-median (Range)	3.00 (1.00, 9.00)
Age groups (years)-no. (no./total no.%)	
<15	23 (8.4)
15–44	191 (69.96)
45–64	47 (17.2)
≥65	12 (4.39)
Antigen detection (positive)- no. (no./total no.%)	102 (86.44%)^b^
Vaccination status- no. (no./total no.%)	
0 dose	25 (9.15)
1 dose	9 (3.29)
2 doses	77 (28.20)
3 doses	162 (59.34)
Source	
Mass testing	3 (1.10)
Risk population^c^	10 (3.66)
Fever clinic	5 (1.83)
Close contact	154 (56.41)
Delimitation-risk areas^d^	99 (36.26)
Controlled zone	62 (22.71)
Lockdown zone	26 (9.52)
Precautionary zone	11 (4.03)

^a^A total of 277 cases were confirmed in this epidemic; only the epidemiology investigation information of 273 cases was accessed by the Guangzhou center of disease control.

^b^There were only 118 patients who had antigen detection.

^c^Those who had a history of traveling to key places during the Omicron epidemic.

^d^Delimitation-risk areas, including the lockdown zone, controlled zone, and precautionary zone.

**Figure 3:**
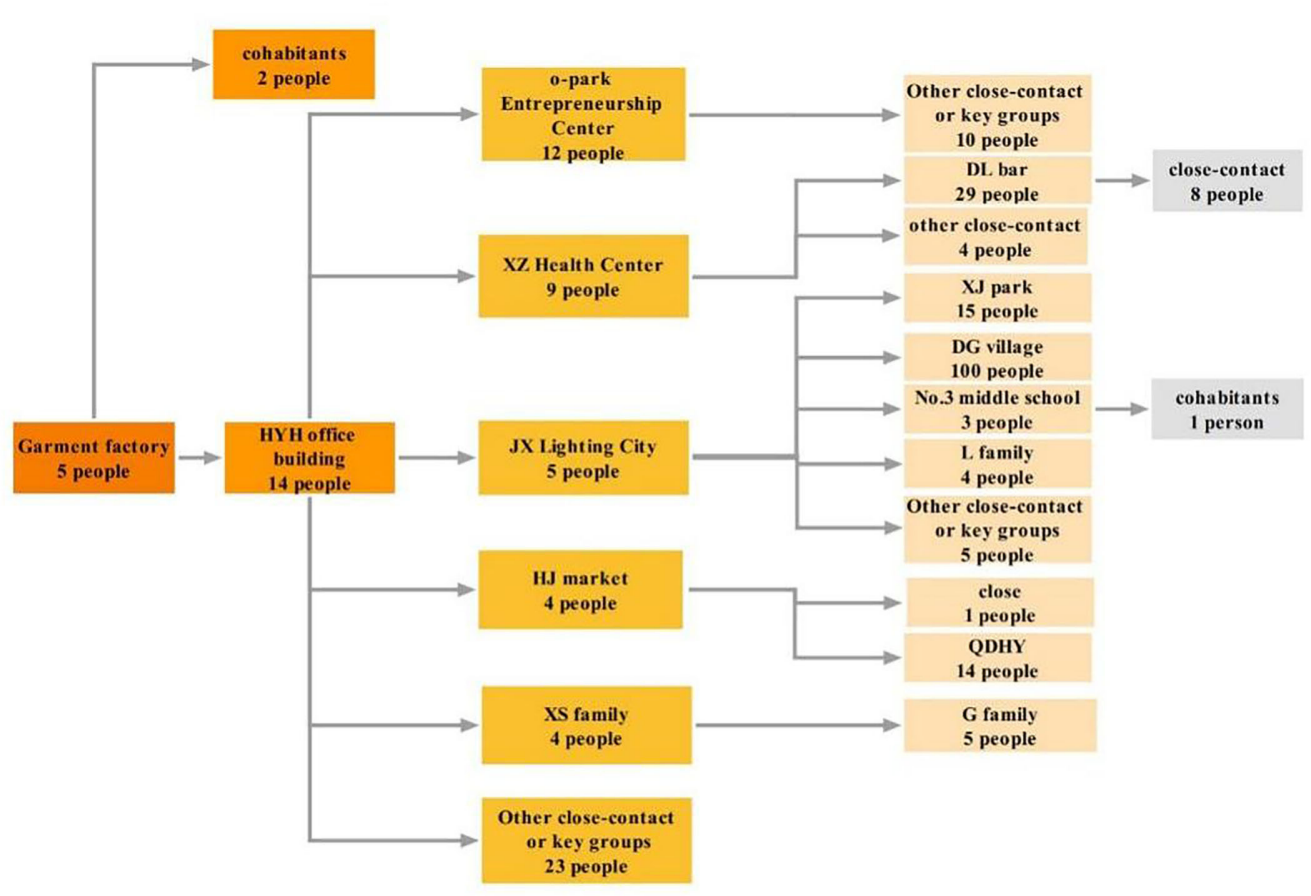
Transmission profile of Omicron epidemic in Guangzhou. Numbers in the figure are based on the results of epidemiology investigation and gene sequencing; the transmission chain was identified with each key place as a single unit, and the different colors represented different generations.

The first case was a 7-year-old student who was identified during a routine back-to-school screening. Subsequently, the government rapidly launched mass testing in each district of Guangzhou, followed by epidemic investigations in key places, dynamic delimitation, and management of at-risk areas. According to the results of the epidemiological survey, this epidemic originated in the YY garment factory and spread to several social entertainment places and subsequently to family members (Figure 3).

### All-out effort to tackle epidemic in key places

**Figure 4:**
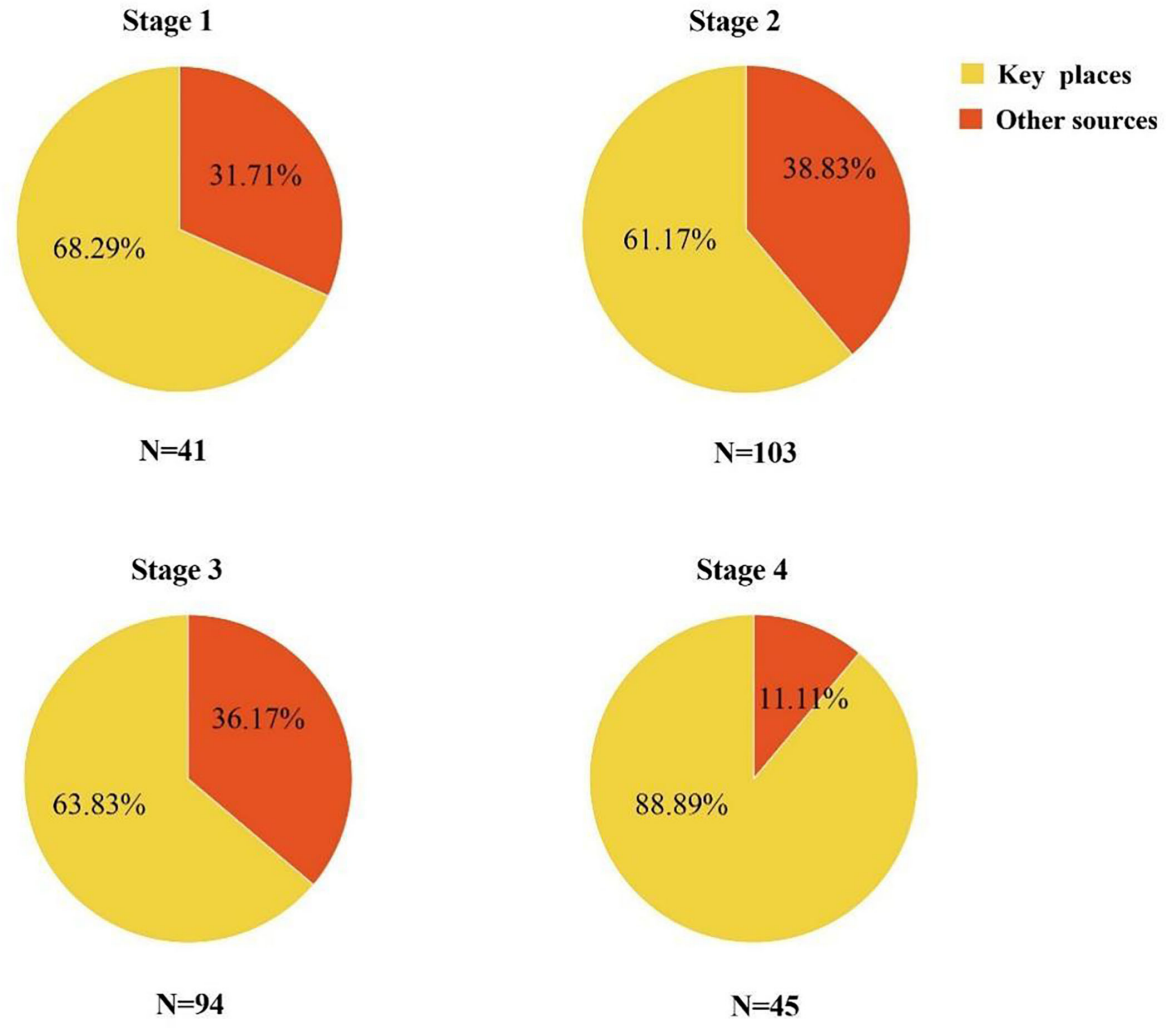
The case-finding source at each stage. Other case-finding sources include mass testing, fever clinic, and close contact.

On the basis of a rapid 24-h epidemic investigation protocol, we had initially designated more than 1,000 key places. Having considered the duration of stay, frequency of activity, information completeness of contacts, and venue ventilation conditions, 15 high-risk key places were identified at last. With the effective management of the key places, the proportion of cases detected increased from 61.17% at Stage 2 to 88.89% at Stage 4 (Figure 4). In addition, parts of key places were located in urban villages (such as the Dagang area), and we adopted the double-cross search pattern (shape of the Union Jack) to screen the neighboring buildings. A total of 26 cases were found in Stage 4, 81% of them in the Dagang area.

### Dynamic delimitation and management of at-risk areas

**Figure 5:**
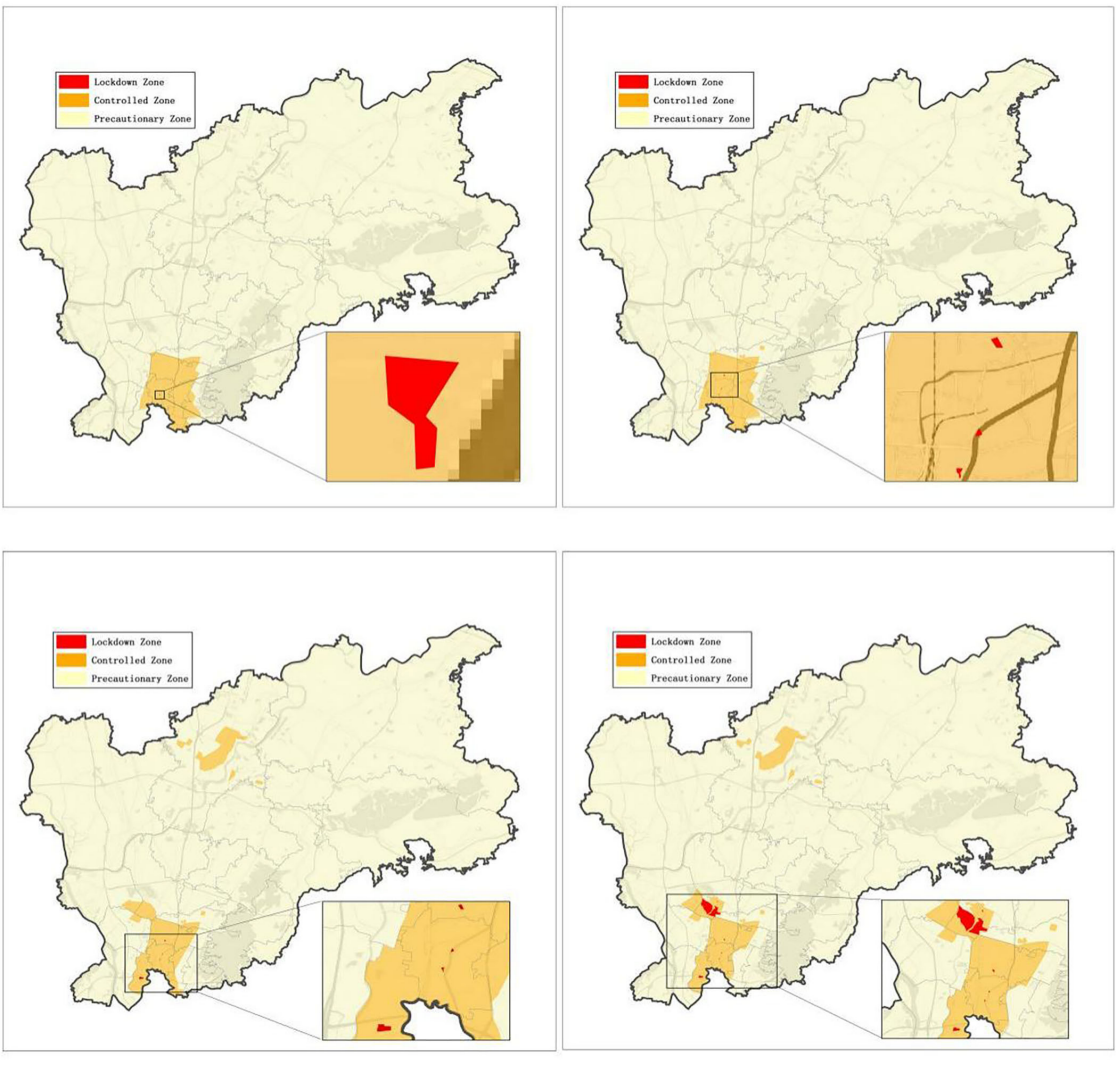
Dynamical delimitation of at-risk zones in Baiyun district.

To block out the transmission chain in a timely manner, we partitioned the at-risk areas from others in the city. Take Baiyun district, for instance, 155 lockdown zones, 71 control zones, and six precautionary zones were delimited throughout the four stages of the epidemic. With the implementation of delimited risk area management (Figure 5), 99 cases were found, and the average interval between a case testing positive and isolation was −2.61 ± 2.76 days in the lockdown zone, −0.44 ± 1.08 days in the controlled zone, and−0.27 ± 0.62 days in the precautionary zone, indicating the transmission risk had been under control in advance since the delimitation of at-risk areas. Also, as illustrated in Figure 5, the range of the lockdown zone and the control zone was dynamically adjusted immediately following the changes in the epidemic situation.

### Health code management of at-risk population

**Figure 6:**
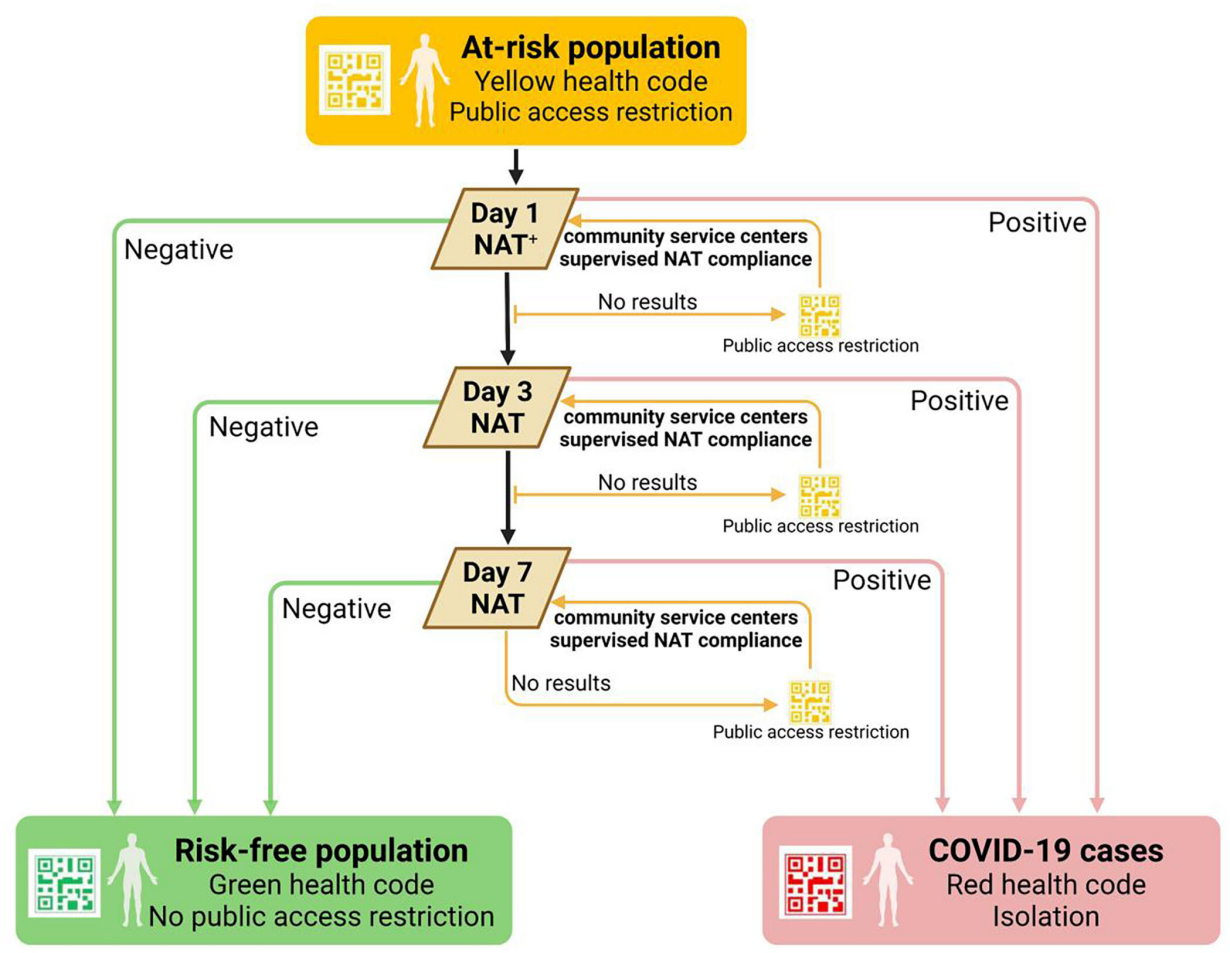
Health code management of the at-risk population. ^+^Public access included public transportation usage and public places entrance. NAT, nucleic acid test.

In order to screen for transmission chains that might have been overlooked, people having spatial–temporal intersections with the cases or absent from nucleic acid screening were assigned yellow health code (Table 1, Figure 6). To eliminate potential risks as quickly as possible, people with yellow code were required to complete the NAT within 24 h after receiving the notification; the staff of community service centers would help to supervise the process. Considering the increased transmissibility of the Omicron variant, the length of NAT monitoring for yellow-coded individuals in this outbreak was extended from 3 to 7 days. People with yellow code were requested to take the NAT on the first, third, and 7^th^ days after receiving the notification. After taking the first NAT, the yellow health code was changed to a green one with a negative result or changed to a red one if a positive result was obtained. However, if people, for some reason, did not take the next detection as requested, the health code would automatically turn back to yellow, urging them to conduct self-health management and take the NAT.

A total of 2,944,484 yellow codes were assigned, of which 84 were eventually confirmed as COVID-19 cases, accounting for 30.32% (84/277). The longest interval between the yellow-code assignment and the date for NAT positive was 9 days; 16.67% (14/84) cases only took 3 days since the yellow code was assigned.

### Effectiveness of epidemic control

**Figure 7:**
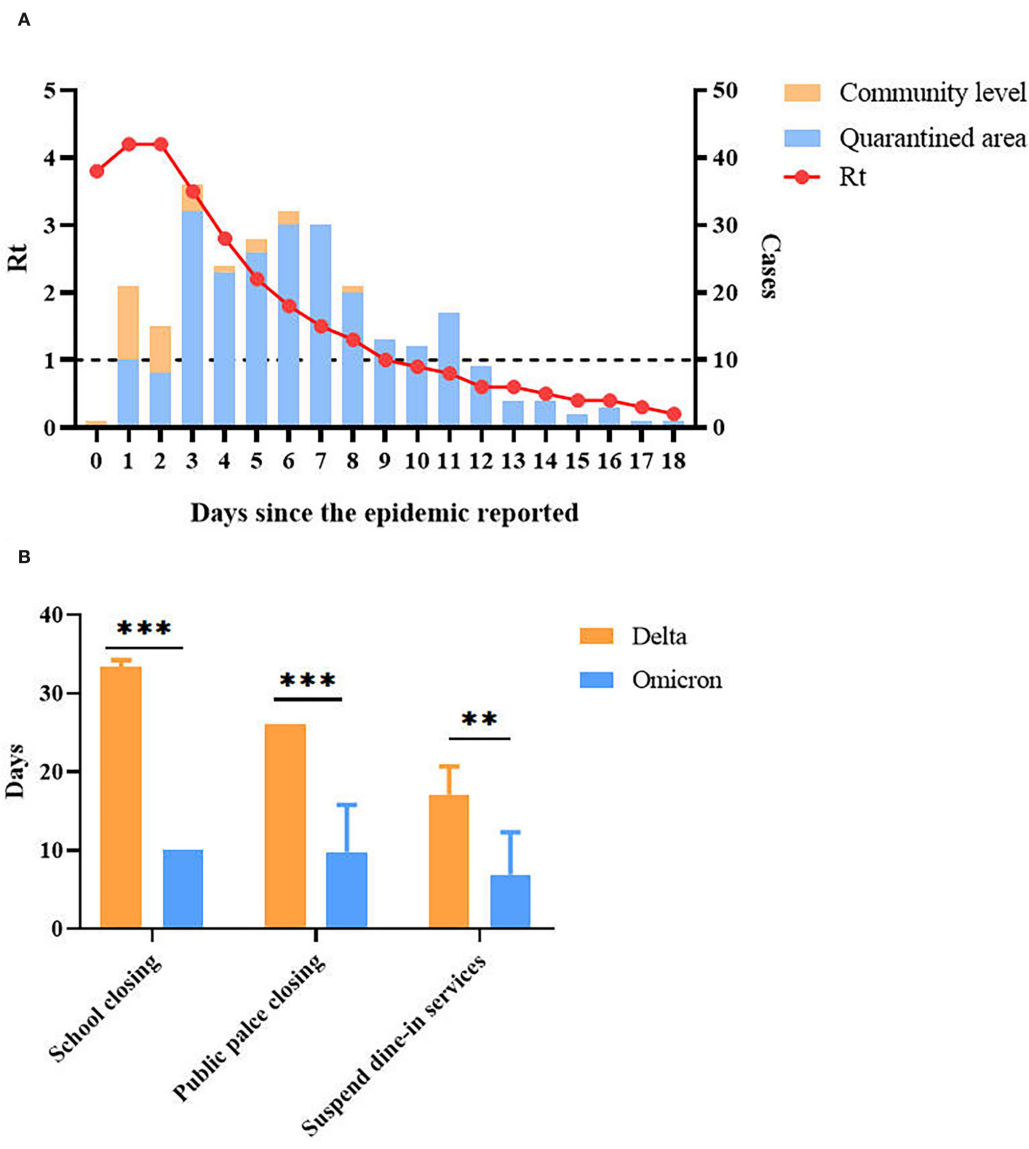
Effectiveness of epidemic control (Omicron vs. Delta). **(A)** Rt throughout the epidemic. The epidemic started date is 8 April 2022. **(B)** The duration of non-pharmaceutical interventions (NPIs) implemented by the government. The ** and *** symbols indicate the *P* values < 0.01 and < 0.001 with the difference between groups are significant respectively.

The effective reproduction number (Rt) rose rapidly at the beginning of this Omicron epidemic, and it reached the peak value (4.20) on the third day since the epidemic began and fell below 1 on the 9^th^ day (6 days later). All COVID-19 transmission chains in communities outside the quarantined areas were cut off after 9 days (Figure 7A).

In addition to evaluating the effectiveness of intervention measures through calculation of Rt, we also compared the duration of non-pharmaceutical intervention policies (NPIs) implemented by the government between the Omicron epidemic and the last year's Delta epidemic, which included school closing, public place closing, and suspended dine-in service, to evaluate the impact on life. As shown in Figure 7B, for the districts outside the epicenter of Guangzhou, the implementation duration of NPIs was much shorter compared with the Delta epidemic last year.

## Discussion

The SARS-CoV-2 Omicron variant has rapidly spread worldwide since November 2021 and become the dominant epidemic strain at present. It is characterized by more mutations, quicker transmissibility, and enhanced immune evasion. Although weakened virulence can be observed in Omicron, the fatalities remained high, especially for the elderly (14, 15). Besides, for older persons, the protective efficacy of two doses of inactivated vaccine immunization is not ideal. It is reported that the effectiveness of the inactivated vaccine (two doses) in preventing serious illness is only 60.7% in those older than 80 years, while an additional booster shot could get a 98% improvement (16). Currently, the percentage of older adults in Guangzhou who have received two doses of the SARS-CoV-2 vaccine is 92.2%, but the percentage of those who received booster shots is only 60.7% (17). Therefore, if the local outbreak of Omicron had not been contained at the beginning, an unpredictable epidemic size would have overwhelmed our medical capacity and disrupted social stability.

In response to this Omicron epidemic in Guangzhou, fast, strict and precise control strategies were implemented. With an all-out effort to tackle the epidemic in key places, dynamic delimitation and management of at-risk areas, and health code management of at-risk populations, the effective reproduction number (Rt) sharply declined, and the implementation of NPIs in non-epicenter was minimized. Remarkably, the Omicron epidemic was contained within 18 days.

In terms of epidemiological characteristics, we observed that the Guangzhou epidemic has the characteristics of rapid spread, strong concealment, and a large number of places involved. First, the median incubation time and generation time were 3 days, which means that the community transmission is quick; second, the first case we found was confirmed to be the third generation or above after tracing the source; and third, the positive cases had obviously clustered in places such as working places, daily living places, and so on. The epidemic mainly originated in the YY garment factory, spread to several social entertainment places, and then spread to homes. More than 87% of the cases had completed two or more doses of inactivated vaccine, which confirmed that Omicron did have the ability to escape immunity to the virus (18, 19).

After the first case was found, we carried out a rapid epidemiological investigation, so as to determine the trajectory of the case in a timely manner, and to delineate key places with a higher risk of the epidemic. Some key places would later be released from control and isolation in time if it was proved that a positive case had not been there within the latent infection period. In order to quickly extinguish the epidemic in key places, we established a work arrangement of “one place, one special group,” which means that police officers, professionals from disease control departments, and staff of community service centers worked on-site together for 24 h a day. Such workflow arrangement guaranteed all the tasks, such as epidemiology investigation, case management and control, transshipment, and isolation in key places run smoothly, thus reducing communication costs, increasing work efficiency, and eventually outpacing the virus transmission.

We defined and divided certain areas with different epidemic risks into three levels (lockdown, control, and precautionary), and each partition had corresponding prevention and control policies with respect to the frequency of NAT and personnel mobility, thus enabling us to better concentrate our efforts and resources on outbreak control. In addition, we subdivided the control zone into 14 control grids according to the specific geographical features (demarcated by adjacent rivers, highways, large buildings, etc.), which was beneficial for the management and delivery of living materials. What is worth mentioning is that the government dispatched staff through the “1 + 5 + 3 + 9 + N” working mechanism to ensure manpower supplement and the normal life of residents in the grids.

Implementation of the yellow health code policy in this Omicron epidemic showed its advantage; it facilitated the identification of at-risk individuals faster and more accurately than that in the Delta outbreak, with approximately 30.32% of COVID-19 cases found among people with yellow health code. The yellow health code daily clearance policy stressed the timely management of the at-risk population. By clearing people holding yellow health code every 24 h, they were urged to complete NAT as soon as possible, thus having CDC professionals work on screening cases in high-risk places. On the other hand, the negative NAT results can be used to redefine a risk-free population and reduce the impact on people's normal life and work. However, in the initial stage of the epidemic, the yellow health code assignment relies heavily on the epidemiological investigation result, which needs several hours to complete. To control the spread as soon as possible, yellow health code management could not take the place of community screening to find out the underlying transmission chain. We suggest NAT screening can be gradually narrowed down to the key population marked with yellow health codes after the community transmission risk is eliminated, reducing its impact brought social and economic development (20).

In total, we summarize the experience in controlling this Omicron epidemic. First, to avoid large-scale outbreaks in the community, routine surveillance of high-risk populations is an indispensable measure for the early discovery of infectious cases; only early initiation of the epidemiological investigation, soon after positive cases are found, can put transmission under control in time. Therefore, it is recommended to establish a routine surveillance workflow on people with high exposure risk. It is evident that fast and accurate epidemiological investigation necessitates key place and population identification, so digital and artificial intelligence tools can be applied to speed up the investigation (21). Moreover, it is extremely important for municipal authorities and professionals from public health institutions to work together to establish and unify all epidemic control measures to conquer the disaster.

What we know about SARS-CoV-2 is only the tip of the iceberg, and its mutation is still ongoing and unpredictable (22). Before the development of more effective drugs and vaccines, in the face of local outbreaks, fast and accurate epidemic prevention and control measures are necessary; the main goal is to reduce the number of infected people for early diagnosis and adequate treatment of confirmed cases, so as to reduce the fatality rate maximally and to guarantee more time for full vaccination of the entire population. However, the way to achieve a balance between epidemic prevention and people living a normal life is an issue that we need to constantly explore.

## Conclusion

In the face of the highly infectious Omicron strain, rapid and comprehensive epidemiological investigation provides assurance of early control of the transmission. Reasonable delimitation of at-risk areas and dynamic refinement of control areas can reduce the impact of epidemic prevention measures on people's lives.

## Data availability statement

The raw data supporting the conclusions of this article will be made available by the authors, without undue reservation.

## Ethics statement

Ethical review and approval was not required for the study on human participants in accordance with the local legislation and institutional requirements.

## Author contributions

ZiY, LZ, NZ conceived the idea of the study. ZM, KM, XK, XL, YaL, QL, CT, CC, CH, PQ, KL, XLi, YM, XLiu, WG, ZZ, WQ, WH contributed to the design of the study and contributing the design of study and data collection. ZiY, NZ, ZhY interpreted the data. WC, ZiY, JL, ZL, YM, CC, YoL contributed to the preparation of the manuscript. All authors contributed to the article and approved the submitted version.
