# Supplementary material for: How fast and how well the Omicron epidemic was curtailed. A Guangzhou experience to share
Source: Front Public Health. 2022 Dec 21;10:979063. doi: 10.3389/fpubh.2022.979063 (PMC9812567; doi:10.3389/fpubh.2022.979063)
Supplement: Supplementary file 1 [file Table_1.docx]

**Table S1. Routine prevention and control measures in non-epidemic period.**

| **Category** | **Subcategory** | | | **Detection frequency** |
| --- | --- | --- | --- | --- |
| **Compulsive nucleic acid testing group** | **Immigrants, close contacts** | | | 1. day centralized isolation, followed by 7-day home health monitoring;   6 nucleic acid and 6 antigen tests were carried out crosswise during this period |
|  | **Employees in high-risk positions** | | | 7 days of centralized isolation, followed by 7 days of home health monitoring;  6 nucleic acid and 6 antigen tests were carried out crosswise during this period |
|  | **Frontliners of pharmacy and Medical Sentinel** | | | 1 test per day |
|  | **None** **front-line employees of medical institutions** | | | 2 tests within7 days |
|  | **Staffs and passengers of intercity and urban public transport** | | | 1 test within 7 days |
|  | **Staffs of Nursing home and prison** | | | No less than 3 tests within 7 days |
|  | **Schools** | **Before school starts:** | School healthcare workers, security, cleaner, dormitory administrators, canteen staffs, express delivery staffs, school bus drivers and staffs on other risk positions | 1 test before returning to work. |
|  |  | **After returning to school:** | Teachers, students, and staffs | 1 test on the returing day |
|  |  | **During the semester:** | Teachers, students, and staff of the school | 1 test per week on a district basis.  During the semester, the division, classification and grading will be randomly checked according to the proportion：  1. In districts that have reported local positive cases in the past 7 days, a sampling check of no less than 20% of the number of teachers and students is completed every day, covering all teachers and students on duty every week.  2. Districts that have not reported local epidemics in the past 7 days will carry out nucleic acid sampling work of on-the-job staff and students every week.  3. For districts that have reported local positive cases in the past 3 months, each district will take a weekly sample of no less than 20% of the total number of teachers, students, and staff in all schools in the district. (Schools with less than 200 students shall have no less than 40 students for each random test).  4. For districts that have not reported local positive cases in the past 3 months, each district should randomly test a sample of no less than 10% of the total number of teachers, students, and staff in all schools in the district each week. (Schools with less than 200 students shall have no less than 20 students for each random test). |
|  | **Construction site workers** | | | At least once a week |
| **Risk industries and risk population groups** | **People from other places returning to Guangzhou** | | | Landing detection at traffic stations |
|  | **Cross-province truck driver returning to Guangzhou** | | | Report 24 hours in advance, scan code for temperature measurement, 48-hour nucleic acid inspection, free nucleic acid testing on the ground, and graded and classified management of vehicles and venues |
|  | **Relevant staffs of primary wholesale markets, large logistics parks and freight stations (including railway freight stations) with relatively concentrated freight vehicle business in Guangzhou from outside the province** | | | 1 test every other day, work with a 48-hour nucleic acid negative certificate |
|  | **Downstream market or park** | | | 2 tests in 7 days, personnel with a 72-hour nucleic acid negative certificate to work |
|  | **Domestic cargo services related staffs** | | | 1 test in 7 days |
|  | **Imported clothing related staffs** | | | Regular nucleic acid testing no less than twice a week |
|  | **Inbound mail, express, and imported goods related staffs** | | | 1 test every other day |
| **Potential risk groups in risk areas** | **Front-line workers of the express delivery industry, sanitation, bars, saunas, farmers markets, restaurants, hotels, cinemas, karaoke, gyms and other indoor-closed and semi-closed places** | | | Nucleic acid testing is carried out every week, and front-line staff will select no less than 25% of the population for nucleic acid testing once a week |
|  | **Front-line workers of public cultural and sports venues, such as tourist attractions, libraries, museums, art galleries, and gymnasiums** | | | Nucleic acid testing is carried out every week, and front-line staff will select no less than 25% of the population for nucleic acid testing once a week |
|  | **Residents of communities or natural villages around risk places such as airports, harbors, railway stations, international health stations, quarantine hotels, and centralized supervision warehouses** | | | A certain number of people are selected every week to carry out nucleic acid screening |
