# Supplementary material for: How fast and how well the Omicron epidemic was curtailed. A Guangzhou experience to share
Source: Front Public Health. 2022 Dec 21;10:979063. doi: 10.3389/fpubh.2022.979063 (PMC9812567; doi:10.3389/fpubh.2022.979063)
Supplement: Supplementary file 2 [file Table_2.docx]

**Table S2. The classification and management of key places**

| **Risk level** | **Principle of risk classification** | **Example of places**  **(Not limited to the following)** |
| --- | --- | --- |
| High risk**^a^** | 1. Places with high activity of COVID-19 cases in 2 days before their symptom onset or the first positive NAT date  2. Places that COVID-19 cases stayed for a long time in 2 days before their symptom onset or the first positive NAT date; and process of close contact, secondary contact determination and identification was time-consuming.  3. If the source of infection is unknown, to identify more potential high-risk places, the time periods can be moved forward from 2 days to more | 1.Residences, workplaces, commercial buildings, industrial parks, etc. 2. Wholesale markets, exhibitions, concerts, supermarkets, funeral homes, nursing homes, hospitals, schools, public transport stations, logistics parks, etc. |
| Medium risk**^b^** | 1. Indoor enclosed places that COVID-19 cases were gathered or dined 2 days before their symptom onset or the first positive NAT date  2. Recreation facilities that COVID-19 cases visited 2 days before their symptom onset or the first positive NAT date | 1. Hotels, KTVs, restaurants, spa and beauty salons, hair salons, gyms, Internet cafes, bath centers, cinemas, chess and card rooms, board game rooms, moxibustion parlors, health centers, video studios, etc. 2. Libraries, museums, tourist attractions, parks, etc. |
| Low risk**^c^** | Places where COVID-19 cases stayed for a short time, with clear trajectories, clear contacts, and contact information can be obtained accurately | Taxi, convenience store, breakfast store, fruit store, temporary store, etc. |

Graded epidemic control for places with different risk levels:

【a】The venue should be closed immediately, and all people in it are not allowed to leave unless completed a nucleic acid test. People with negative NAT will be subsequently graded managed. After collecting environment samples, the venue is completely sterilized and strictly closed for no less than 7 days.

【b】The venue should be closed immediately, and all people in it are not allowed to leave unless completed a nucleic acid test. People with negative NAT will be subsequently graded managed. After collecting environment samples, the venue is completely sterilized and strictly closed for no less than 7 days. When the venue meets the following conditions, it can be reported by local prevention and control office for approval of lifting the closed management or shortening the closed period: 1) negative NAT of environment samples, 2) environmental risk factors could be excluded, 3) close contacts and secondary close contact investigations have been screened, 4) and a low follow-up risk is conclude by on-site teams.

【c】The venue will be closed for 1-3 days and re-opened when the following conditions are met 1) investigation and management of close contacts and secondary close contacts are completed, 2) employees and other at-risk people have complete NAT , 3) environment samples are NAT negative after terminal disinfection, 4) and the environmental risk factors have been excluded.
